# Prostate Cancer Support Groups: The Unadvertised Camaraderie

**DOI:** 10.7759/cureus.18208

**Published:** 2021-09-23

**Authors:** Shana Santarelli, Nicole Ambrose, Zachariah Taylor, Paulette Dreher, Noah May

**Affiliations:** 1 Urology, Philadelphia College of Osteopathic Medicine, Philadelphia, USA; 2 Urology, Main Line Health, Philadelphia, USA

**Keywords:** prostate cancer effects, prostate cancer psychosocial burden, cancer support groups, prostate cancer (pca), prostate cancer support groups (pcsg)

## Abstract

Prostate cancer (PCa), in particular, is known to cause significant psychosocial distress during the duration of a patient’s treatment due to its uncertainty and demasculinizing side effects. Prostate cancer support groups (PCSGs) have been proven to be beneficial, yet are underutilized by the majority of PCa patients and physicians. A thorough review of the literature was performed for articles pertaining to prostate cancer support groups. We sought to identify factors contributing to the psychological burden of the disease, factors that influenced patients to join, and barriers to participation in a PCSG. Additionally, the characteristics and format of PCSGs, as well as outcomes (i.e. quality of life), were evaluated.

## Introduction and background

Prostate cancer (PCa) is the most common cancer in men across 112 countries, with 1.4 million new diagnoses in 2020 according to the GLOBOCAN database, leading to an incidence of PCa approaching 38 per 100,000 men [1]. Advances in PCa therapies have greatly improved overall survival in men with PCa over the past 20 years [2]. However, 30-50% of men with PCa report a psychosocial burden due to their diagnosis regardless of disease stage or progression [3]. As such, the psychological aspects of a PCa diagnosis have become an important part of the complete treatment plan.

Common psychosocial issues include anxiety, depression, as well as fear of imminent (prostate-specific antigen) PSA assessments, potential recurrence of disease, and the possibility of death. In addition, PCa is unique in that it also affects a patient’s sexual function, further exacerbating the psychosocial burdens [4]. In light of these PCa specific stressors, the need and motivation for utilization of prostate cancer support groups (PCSGs) could be better understood. While literature describes an unmet need for support, PCSGs remain an underutilized tool in aiding men with a PCa diagnosis [5]. In this review, we aim to describe the role of PCSGs in the treatment of PCa and its documented benefits in the hope of promoting more consistent development and utilization of these services.

## Review

Methods and results

A comprehensive literature review search was performed using PubMed, SciELO, ScienceDirect, and Google Scholar for relevant English articles published in the past 10 years. Free text search terms included, “prostate cancer support groups,” “cancer support groups,” “psychological burden of prostate cancer,” “online support groups,” “prostate cancer treatment side effects”. Each article was carefully analyzed for appropriateness for inclusion giving preference to the last 10 years by three reviewers into this present discussion.

We found 23 relevant articles in our search, giving precedence to the last 10 years. The most relevant articles were retrospective studies or surveys sent to patients participating in PCSGs. The most utilized questionnaire was Flanagan’s Quality of Life (QoL) Scale. 

Financial burden, treatment side effects, and psychological implications of prostate cancer

The burden of prostate cancer is not necessarily dictated by the aggressiveness or stage of the disease. Compounding factors such as a new cancer diagnosis, financial stress of treatment, and the physical and psychological repercussions of treatment contribute to a complicated and significant burden for the patient. In a 2018 study, the median cost per patient within three years following prostate cancer diagnosis was $14,452, with treatment costs accounting for the majority ($10,558) [6]. PCa treatment also often results in significant side effects such as erectile dysfunction, loss of libido, urinary and/or bowel incontinence. Distinct from other cancers, the cancer diagnosis of PCa has been shown to dramatically increase the risk of a psychiatric syndrome, with greater than 30% of PCa patients reporting a new psychiatric diagnosis [7]. Up to 30-40% of these patients admitted that the anxiety associated with their cancer diagnosis affected their daily living [5]. In a prospective clinical trial, Roumier et al. demonstrated that PSA testing is particularly anxiety-inducing and was one of the reasons PCa patients fell out of surveillance [8]. Depression in PCa patients has been attributed to the feeling of “lack of manliness” and poor sexual performance and is often underappreciated in PCa patients [9]. Importantly, depression has been associated with worse survival in PCa patients. It is proposed that treatment of depression improves a patient’s health maintenance behavior, health care utilization, and immune function [10]. Although participation in PCSG has not been directly shown to affect overall survival or cancer survival, it could be postulated that it does increase overall and cancer survival since depression treatment helps the immune function. The burden of PCa diagnosis and treatment is complicated and multi-factorial, as such there is an ever-growing need for the development, promotion, and utilization of PCSGs.

History of cancer support groups and PCSGs

Psychosocial support groups have been implemented in patient-care following a cancer diagnosis over the last two decades and have been shown to improve psychological wellbeing, reduce anxiety and depression, and provide the patient with better coping abilities and mental adjustments [11]. Previous qualitative studies demonstrated many positive outcomes associated with attending cancer-specific support groups by providing a unique sense of community, unconditional acceptance, accurate cancer-specific information, and personal empowerment on the contrary to the isolation and rejection individuals can feel independently [11-13]. 

Approximately, one in four cancer survivors utilize support groups, but only 10.2% of patients report that their physician recommended a support group [13]. Support groups have been successful with many cancers, including breast and colorectal [14-15]. Among cancer diagnoses, breast cancer and PCa support groups are the most sought out by patients because of the “surrounding stigma” of the disease [16]. “Surrounding stigma” is associated with a diagnosis where those affected are looked upon in an inferior way, previously seen in HIV or AIDS for example [16].

Dunn et, al. (2017) interviewed 21 physicians in Australia who lead PCSG, the site of many early PCSGs, to identify their motivations for developing these groups. They reported their goal was to help men’s feelings of isolation due to their PCa diagnosis and treatment [17]. A Canadian survey conducted by Oliffe, et al. (2015) concluded that PCSGs were particularly appreciated by specialists, including urologists and oncologists, for their ability to share information and provide psychological support particularly for in-person groups [18]. Despite the fact that PCSGs have been consistently appreciated by healthcare providers, PCSG are not universally recommended or available. 

Distribution of PCSGs in the United States (US) are not readily available. However, The Prostate Cancer Foundations offers a Support Group tab on their website that allows patients to find several organizations [19]. These organizations include: "Us, Too - National", "Us, Too - Local", "His Prostate Cancer- support for partners", "Male Care", and "Imerman Angels" [19]. 

Structure of PCSGs

PCSGs exist in various iterations to aid in ease of access and efficacy for the participants. Both in-person and online support groups have been reported in the literature. PCSGs are designed to aid in coping with diagnosis, management of treatment side effects, and recovery. One study reported exercise facilitated counseling where patients do an exercise regimen then meet and debrief about their diagnosis [20]. 

A cross-sectional comparison showed individuals utilizing face-to-face groups have a better capacity for exchanging information, gaining recognition, and caring for others [21]. Online support group members tend to be an average of six years younger than those who seek face-to-face support [22]. Concerns for online forums included the inability to captivate men with PCa and the lack of inclusivity, privacy, and accessibility. Although online support groups are easily accessible to anyone with access to the internet, it is important to be aware of their barriers. In the era of an international pandemic, online groups may become more prominent, but it is important to recognize that there may be a benefit for some men to attend an in-person group depending on the preference and needs of each individual.

PCSG participation and benefits

Who Seeks PCSGs?

**Figure 1:**
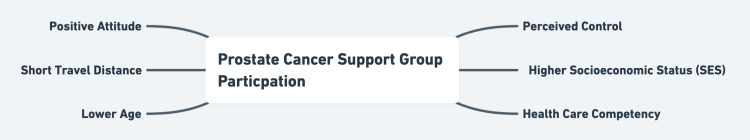
Prostate Cancer Support Group Participation

In a group of 438 PCa Patients, Voermann, et al. (2007) assessed predictors of intention and actual participation in support groups and found that a more positive attitude and perceived control are the strongest predictors of interest in group participation. Additionally, patients with lower age, higher socioeconomic status (SES), and lack of social support are also more likely to join a support group as shown in Figure 1 [23]. A cross-sectional study conducted by Haack, et al. (2018) discovered that a patient who was noted to have competency navigating through a healthcare system is 1.8 times more likely to join a PCSG [24]. Men have been found to join PCSG at specific points in their disease process. Adikari, et al. showed PCa patients join online support groups at four specific psychological phases: diagnosis, treatment, side-effects, and recurrence, with 67% of patients joining at the time of treatment initiation [25]. Carter, et al. (2011) reported patients with advanced PCa are more likely to seek PCSGs and desire stage-specific information to address concerns about the diagnosis [26]. 

Benefits of PCSGs

In 2011, Bestmann, et al. created the HAROW project, a retrospective German study, to assess the quality of life for 504 men in PCSGs. Overall a high QoL was rated in PCa patients enrolled in PCSGs but was dependent on their treatment plan. Combined hormonal therapy and radiation therapy reported a lower QoL, while patients with cured tumors report a higher QoL [27]. The HAROW project demonstrated that PCSG patients rate their QoL very similar to a person under normal health circumstances [27]. In addition, the Prostate Cancer Peer Support Inventory (PCSI) was a survey conducted on participants in 44 different PCSGs assessing their QoL, psychological distress, inconvenience from pain and tiredness, and perception of clinician support within support groups. Overall, peer support in PCSGs was rated high throughout. Younger age, lower pain, lower education, better attendance, use of alternative therapies, higher perceived clinician support, and reported higher QoL was associated with a higher satisfaction with PCSGs [28]. Psychological distress for patients in PCSG was also low with only 8% of participants indicating high psychological distress that would be consistent with a post-traumatic stress disorder diagnosis [29]. This is significant because the incidence of the full syndrome of post-traumatic stress disorder can reach up to 22% in cancer survivors evaluated after treatment [29]. An online survey by Capistrant, et al. (2018), targeting gay and bisexual men specifically, showed PCSGs to be uniquely beneficial, in that information and emotional support were better received when these men were in attendance [30]. Adikari et al, demonstrated that patients joining support groups pre-treatment versus post-treatment had fewer negative emotions after participating in support groups and had an improvement in overall emotional well-being [27]. Overall, PCSGs provide improved QoL at multiple stages in a patient's treatment regimen and decreased risk of post-traumatic stress disorder following treatment. 

Future directions of PCSGs

Unfortunately, there are minimal studies that show direct benefits of PCSGs. Randomized controlled studies (RCTs) that not only compare QoL but survival benefits of PCSG participants would be more valuable and future studies should incorporate this. RCTs would give providers more substantial information, which they can present to patients when presenting these groups. Having providers act as the advertisers of these groups at clinics would eliminate the lack of awareness of PCSGs among PCa patients who might be longing for support. 

## Conclusions

PCSGs have been proven to improve QoL in patients diagnosed with PCa, particularly if participation is established prior to treatment initiation. Despite this, attendance is poor and PCSGs are dramatically underutilized within the community. Poor promotion or lack of awareness of PCSGs by physicians and healthcare workers is likely the leading cause of lack of participation among PCa patients. Online and in-person groups have both proven to be beneficial and physicians should provide patients with multiple options to ensure necessary access, comfort, and needs of the patient are met.
